# Évaluation du niveau de préparation des districts de santé de la région du Littoral, Cameroun face à une éventuelle épidémie de mpox

**DOI:** 10.11604/pamj.supp.2025.50.1.45474

**Published:** 2025-04-11

**Authors:** Alex Stéphane Ndjip Ndjock, Steve Roland Souga, Michaela Josee Meli, Alice Ketchaji, Hans Makembe Mossi, Ingrid Cecile Djuikoue, Camille Paulin Franc Ntolo, Ernest Tambo, Andre Bita Fouda

**Affiliations:** 1 District de Santé d´Edea, Région du Littoral, Douala, Cameroun,; 2 Association pour le Développement de L’épidémiologie de Terrain, Château de Vaccassy, 12, Rue du Val d’Osne, 94415 Saint-Maurice Cedex, France,; 3 Faculté des Sciences de la Santé, Université des Montagnes, Bangangté, Cameroun,; 4 Cameroon Society of Epidemiology, P.O Box 1411, Yaoundé, Cameroun,; 5 Faculté de Médecine et des Sciences Pharmaceutiques, Université de Dschang, Dschang, Cameroun,; 6 Direction de la Lutte contre la Maladie, des Epidémies et des Pandémies, Ministère de la Santé Publique, Yaoundé, Cameroun,; 7 Centre Régional de Prévention et de Lutte Contre les Epidémies et les Pandémies, Délégation Régionale de la Santé Publique du Littoral, Douala, Cameroun,; 8 Prevention and Control Foundation, Yaounde, Cameroon,; 9 Département des Sciences Appliquées à la Santé, Institut Universitaire et Stratégique de l´Estuaire, Douala, Cameroun,; 10 Centre for Leadership in Global Health, University of Global Health Equity, Kigali, Rwanda,; 11 Faculté de Médecine et des Sciences Pharmaceutiques, Université de Douala, Douala, Cameroun

**Keywords:** Evaluation, districts de santé, Littoral, Cameroun, epidémie, mpox, Assessment, health districts, Littoral Region, Cameroon, epidemic, mpox

## Abstract

L’épidémie de mpox qui s’est propagée à l’échelle mondiale en 2022 a mis en lumière l’importance cruciale de la préparation aux urgences de santé publique. Bien que le mpox ne soit pas une nouvelle maladie, son apparition soudaine et son expansion rapide dans de nombreux pays tels que le Cameroun a soulevé de nombreuses questions sur la capacité des systèmes de santé à y faire face. Cette étude avait pour objectif d´évaluer le niveau de préparation des districts de santé de la région du Littoral au Cameroun face à une éventuelle épidémie de mpox, afin d’identifier les forces, les faiblesses et les domaines prioritaires d’intervention. Il a été mené une étude descriptive transversale sur une période allant d´aout à septembre 2024, dans les districts de santé de la région du Littoral au Cameroun. Une grille d’évaluation de la préparation face à une éventuelle épidémie de mpox a été élaborée et administrée auprès des responsables des équipes cadres des districts, basée sur le guide technique de Surveillance Intégrée des Maladies et Riposte (SIMR). Les données collectées ont été analysées avec le logiciel Excel 2016. Au total 17 districts ont été enquêtés. En rapport avec le niveau de préparation, il ressort que 5 districts (29%) avaient un niveau de préparation insuffisant (Nkondjock, Loum, Manoka, Ndom, Pouma); 3 districts (17%) avaient une préparation limitée (Boko, Manjo, Dibombari); 7 districts (41%) avaient un niveau de préparation moyen (Bangue, Edea, Logbaba, Ngambe, Yabassi, Njombe Penja, Nkongsamba) et seuls 2 districts (11%) avaient un bon niveau de préparation (Newbell, Mbanga). Aucun district de la région n´avait le niveau de préparation optimal. Aussi, il a été observé de grandes disparités entre les districts avec des scores variants de 0,16 (Nkondjock) à 3,21 (Newbell). La surveillance épidémiologique et laboratoire était la plus significative dans la région avec le meilleur niveau de préparation (préparation Moyenne) (3,01) à l´opposé de la prise en charge des cas (préparation insuffisante) (1,07). Les principaux obstacles à la préparation étaient l´absence d’un plan de préparation de riposte au mpox, l´allocation insuffisante de ressources financières et matérielles, les capacités insuffisantes de diagnostic et de confirmation en laboratoire, l´indisponibilité de protocole de prise en charge clinique, le déficit de formation du personnel de santé sur la prise en charge et l´absence d’un plan de communication des risques. Les districts de santé de la région du Littoral avaient de façon générale un niveau de préparation limité. Plusieurs obstacles pouvant entraver la préparation à la riposte ont été identifiés. Afin de prévenir une éventuelle épidémie de mpox dans les districts de santé de la région du Littoral, il est essentiel de renforcer la préparation des districts sur la coordination et gestion de la réponse, la prise en charge des cas, la communication des risques et engagement communautaire, les mesures de prévention et de contrôle en intégrant une approche différenciée tenant compte des disparités entre districts.

## Abstract (en)

The 2022 global mpox outbreak highlighted the critical importance of public health emergency preparedness. Although mpox is not a new disease, its sudden emergence and rapid spread in many countries, including Cameroon, raised numerous concerns about the ability of health systems to respond effectively. This study aimed to assess the level of preparedness of health districts in the Littoral Region of Cameroon in the event of a potential mpox outbreak, to identify strengths, weaknesses, and priority areas for intervention. We conducted a descriptive cross-sectional study in the health districts of the Littoral Region of Cameroon from August to September 2024. A grid for evaluating preparedness for a potential mpox outbreak was developed and administered to the district health management teams. This tool was based on the Integrated Disease Surveillance and Response (IDSR) technical guidelines. Data collected were analyzed using Microsoft Excel 2016. A total of 17 health districts were surveyed. Regarding their level of preparedness, 5 districts (29%) showed insufficient preparedness (Nkondjock, Loum, Manoka, Ndom, Pouma); 3 districts (17%) had limited preparedness (Boko, Manjo, Dibombari); 7 districts (41%) had an average level of preparedness (Bangue, Edea, Logbaba, Ngambe, Yabassi, Njombe Penja, Nkongsamba); and only 2 districts (11%) demonstrated good preparedness (Newbell, Mbanga). No district in the region reached the optimal level of preparedness. Significant disparities were observed among the districts, with preparedness scores ranging from 0.16 (Nkondjock) to 3.21 (Newbell). Epidemiological surveillance and laboratory capacity had the highest preparedness level (average preparedness score of 3.01), in contrast to case management (insufficient preparedness score of 1.07). The main barriers to preparedness included the absence of a specific mpox response plan, inadequate allocation of financial and material resources, insufficient diagnostic and laboratory confirmation capacity, lack of clinical management protocols, limited training of health personnel on case management, and absence of a risk communication plan. The health districts in the Littoral Region of Cameroon generally exhibited a limited level of preparedness. Several obstacles to preparedness were identified. To prevent a potential mpox outbreak, it is essential to strengthen district-level preparedness, particularly in coordination and response management, case management, risk communication and community engagement, and prevention and control measures, while adopting a differentiated approach that accounts for disparities between districts.

## Introduction

La variole du singe a récemment été déclarée comme urgence de santé publique de portée internationale par L´Organisation mondiale de la Santé (OMS) et *Africa Centers for Disease Control and Prevention (CDC)* avec plus de 185 pays, inclut l´Afrique ayant eu entre janvier 2022 et juillet 2024, 15 États membres de l’Union Africaine qui ont collectivement signalé 37 583 cas et 1 451 décès. En 2024, la République démocratique du Congo (RDC) s’est imposée comme l’épicentre de l’épidémie, concentrant à elle seule 96,3% des cas et 97% des décès. Cette année, le nombre de cas et de décès a respectivement augmenté de 160% et 19% par rapport à la même période en 2023 [1,2].

La variole du singe est une zoonose dont les symptômes ressemblent à ceux de la variole et qui est causée par un virus appelé virus de la variole du singe. Elle émerge et réémerge continuellement en Afrique, le premier cas humain ayant été observé en 1970 en RDC chez un bébé de 9 mois suspecté d’avoir contracté la variole. De ce fait, ce virus est caractérisé par deux classes génétiques: le clade I en Afrique centrale et le clade II en Afrique de l´ouest; seul le Cameroun a signalé la présence des deux clades [3-5]. La fièvre, la gêne musculaire, les maux de tête, l’épuisement et les problèmes respiratoires sont des symptômes précoces courants de l’infection par le virus de la variole du singe. Ce virus se propage de diverses manières, notamment par la consommation de viande, les contacts interhumains, en particulier par l’intermédiaire de lésions cutanées contagieuses dans des endroits tels que les organes génitaux et la bouche, ainsi que par contact direct avec des animaux infectés [6]. La variole du singe n’a pas été signalée en dehors de l’Afrique jusqu’en 2003, lorsqu’une épidémie de 47 cas confirmés ou probables s’est produite aux États-Unis à la suite d’une exposition à des chiens de prairie de compagnie infectés. Ces dernières années, plusieurs cas de variole du singe associés à des voyages ont été recensés, tous à la suite d’expositions au Nigeria [7,8]. Avant 2022, le Cameroun n’avait enregistré que 4 cas isolés de variole du singe, soit un cas par décennie environ. L’année 2024 a été marquée par une recrudescence significative de la maladie, avec 30 cas suspects dont 5 confirmés et 2 décès, principalement concentrés dans les régions du Sud-Ouest et du Nord-Ouest. Les régions du Littoral, du Centre et de l’Est ont également été touchées au cours des dernières années [9,10].

L’identification des maladies infectieuses émergentes et réémergentes nécessite un système efficace. Pour éviter que l’épidémie de variole du singe ne se transforme en pandémie, six interventions fondamentales doivent être mises en œuvre, parmi lesquelles la prévention et le contrôle des infections, ainsi que le partage rapide des données et la communication sur les risques [11]. La variole du singe n’est pas nouvelle, mais elle est rare: il y a eu très peu de cas avant 2022, et presque tous en Afrique de l’ouest et en Afrique centrale. Ce fait, combiné aux faibles taux de mortalité, a entraîné une faible demande de contre-mesures médicales [12]. De nombreux pays ont tiré des leçons et expériences de la pandémie de coronavirus (COVID-19) en développant leur système de santé d´urgence et leurs mécanismes de réponse. L’épidémie actuelle de variole du singe se caractérisant par des manifestations cliniques atypiques et une transmission interhumaine rapide, les mesures de santé publique visant à contrôler les sources d’infection, à bloquer les voies de transmission et à protéger les populations vulnérables sont d’une importance capitale [13]. Cependant il existe très peu d´informations sur la préparation et les mécanismes mises sur place afin de prévenir et répondre aux urgences sanitaires. C’est dans ce contexte que notre étude vise à évaluer le niveau de préparation des districts de santé de la région du Littoral au Cameroun, afin d’identifier les forces et les faiblesses du système de santé local face à cette menace.

## Méthodes

**Type d´étude:** il s´agissait d´une étude descriptive transversale menée du mois d´aout au mois de septembre 2024 dans 17 districts de santé de la région du Littoral, Cameroun.

**Sites d´étude, échantillonnage et population:** l´étude a été menée auprès des districts de santé de la région du Littoral, Cameroun (17) ayant accepté de participer à l´étude en répondant à la grille d´évaluation. Il s´agissait des districts de santé de Bangue, Boko, Edea, Logbaba, Manoka, Ndom, Ngambe, Pouma, Yabassi, Manjo, Newbell, Njombe Penja, Mbanga, Nkondjock, Loum, Nkongsamba, Dibombari.

**Sources de données:** une suite de sources a été recensée pour améliorer la collecte compréhensive des données épidémiologiques provenant des systèmes de surveillance nationaux et internationaux, l´examen des lignes directrices, protocoles et recommandations au sein des districts de santé, la revue de la littérature scientifique pertinente (articles de recherche, rapports d’évaluation) et la collecte d’informations auprès des responsables des équipes cadres des districts

**Collecte de données:** L´étude a utilisé une grille d’évaluation de la préparation face à la riposte de l’épidémie de mpox, basée sur le guide technique de Surveillance Intégrée des Maladies et Riposte (SIMR). Elle a été administrée auprès des responsables des équipes cadres des districts afin de favoriser l’appropriation et l’engagement dans les efforts d’amélioration. La notation utilisée dans la grille d’évaluation de la préparation face à la riposte de l’épidémie de mpox était de type “échelle de Likert” à 6 niveaux (de 0 à 5) et permettait de quantifier le niveau de préparation pour chaque composante évaluée, allant de “Pas de préparation” à “Préparation optimale” (0 = Pas de préparation, 1 = Préparation limitée, 2 = Préparation partielle, 3 = Préparation moyenne, 4 = Bonne préparation, 5 = Préparation optimale). Chaque chiffre ayant une signification bien précise (Tableau 1).

**Tableau 1:** échelle de Likert et interprétation du score moyen obtenu selon l’échelle de Likert

Score	Signification
0	Pas de préparation: aucune mesure, plan ou capacité en place pour cette composante.
1	Préparation limitée: Quelques éléments de base en place, mais des lacunes importantes.
2	Préparation partielle: des éléments clés sont en place, mais des améliorations substantielles sont nécessaires.
3	Préparation moyenne: la majorité des éléments essentiels sont en place, avec quelques points à renforcer.
4	Bonne préparation: tous les éléments essentiels sont en place et fonctionnent de manière adéquate.
5	Préparation optimale: tous les éléments essentiels sont en place et fonctionnent de manière optimale, avec une marge de progression limitée.
**Interprétation du score moyen obtenu selon l'échelle de Likert**	
**Score moyen**	**Niveau de préparation**
0 à 1	Préparation insuffisante
1,1 à 2	Préparation limitée
2,1 à 3	Préparation moyenne
3,1 à 4	Bonne préparation
4,1 à 5	Préparation optimale

Cette échelle ordinale permettait d’évaluer le niveau de préparation pour chaque composante clé de la riposte au mpox. Un score de 3 ou plus indiquait une préparation satisfaisante, tandis qu’un score inférieur à 3 signalait des lacunes à combler rapidement. Elle offrait l’avantage de fournir une évaluation nuancée et semi-quantitative, plutôt qu’une simple réponse binaire “oui/non”. Ceci permettait d´identifier plus précisément les forces et les faiblesses dans la préparation à la riposte contre le mpox en fonction de chaque composante à la suite d´une interprétation du score moyen obtenu selon l’échelle de Likert (Tableau 1).

**Analyse des données de la préparation:** Une analyse par composante des scores des grilles d´évaluation avec Microsoft Excel 2016, couplée à une évaluation comparative avec des références ou des normes établies. Cinq (5) composantes clés incluaient: coordination et gestion de la réponse, surveillance épidémiologique et laboratoire, prise en charge des cas, communication des risques et engagement communautaire, mesures de prévention et de contrôle. Chaque composante était évaluée à la fin par un score moyen. Ensuite, le score global moyen de préparation par district était calculé à l´aide de Microsoft Excel 2016. A la suite, les lacunes et des domaines prioritaires d’intervention étaient identifiés.

**Considérations éthiques:** cette étude est conforme à la déclaration d’Helsinki. Aucune approbation n’a été requise.

## Résultats

**Surveillance épidémiologique et laboratoire:** la surveillance épidémiologique et laboratoire était la plus significative dans la région avec le meilleur niveau de préparation (préparation moyenne) (3,01). Les capacités de diagnostic et de confirmation en laboratoire restaient l´élément le plus faible avec une préparation limitée, tandis que les éléments analysés et l’utilisation des données de surveillance; la définition de cas standardisés et partagés; avaient un bon niveau de préparation. Uniquement 3 districts (17%) avaient un niveau de préparation moyen; 29% (5/17) avaient un bon niveau de préparation et 23% (4/17) avaient un niveau de préparation optimale soient les districts de santé de Bangue, Manjo, Newbell et Nkongsamba. Le système de notification et de remontée des cas était l´élément le mieux préparé (bonne préparation) dans la région du Littoral toutes dimensions confondues (Tableau 2).

**Tableau 2:** surveillance épidémiologique et laboratoire

	Bangue	Boko	Edea	Logbaba	Manoka	Ndom	Ngambe	Pouma	Yabassi	Manjo	Newbell	Njombe penja	Mbanga	Nkondjock	Loum	Dibombari	Nkongsamba	Littoral
**Surveillance épidémiologique et laboratoire**																		
Définition de cas standardisée et partagée	3,00	4,00	5,00	3,00	3,00	0,00	3,00	0,00	4,00	5,00	5,00	5,00	5,00	0,00	3,00	2,00	5,00	3,24
Système de notification et de remontée des cas	5,00	4,00	5,00	2,00	2,00	3,00	5,00	2,00	4,00	5,00	5,00	5,00	5,00	0,00	1,00	4,00	5,00	3,65
Capacités de diagnostic et de confirmation en laboratoire	5,00	0,00	0,00	3,00	0,00	0,00	4,00	0,00	2,00	3,00	2,00	2,00	2,00	1,00	0,00	1,00	2,00	1,59
Analyse et utilisation des données de surveillance	5,00	4,00	5,00	3,00	2,00	5,00	4,00	2,00	3,00	4,00	5,00	3,00	4,00	1,00	2,00	4,00	5,00	3,59
Score moyen	4,50	3,00	3,75	2,75	1,75	2,00	4,00	1,00	3,25	4,25	4,25	3,75	4,00	0,50	1,50	2,75	4,25	3,01

**Communication des risques et engagement communautaire:** l´engagement communautaire et la communication des risques étaient la deuxième dimension en termes de préparation contre le mpox dans toute la région du littoral (Préparation limitée). Huit districts (47%) avaient un niveau de préparation moyen. Seuls 11% (2/17) avaient un bon niveau de préparation (Edea, Mbanga); tous les autres districts avaient de faibles niveaux de préparation allant du niveau insuffisant (35%) au niveau limité (5%). Aucun district n´avait un niveau de préparation optimal. Les éléments mécanismes de dialogue et de participation communautaire et diffusion d’informations claires et adaptées avaient les meilleurs niveaux de préparation (préparation moyenne); tandis que le niveau de préparation de l´élaboration d’un plan de communication des risques restait limité (Tableau 3).

**Tableau 3:** communication des risques et engagement communautaire

	Bangue	Boko	Edea	Logbaba	Manoka	Ndom	Ngambe	Pouma	Yabassi	Manjo	Newbell	Njombe penja	Mbanga	Nkondjock	Loum	Dibombari	Nkongsamba	Littoral
**Communication des risques et engagement communautaire**																		
Élaboration d'un plan de communication des risques	0,00	0,00	4,00	2,00	0,00	2,00	3,00	2,00	3,00	0,00	3,00	1,00	3,00	0,00	0,00	2,00	1,00	1,53
Identification et mobilisation des parties prenantes	2,00	0,00	3,00	3,00	1,00	2,00	3,00	3,00	3,00	0,00	2,00	2,00	3,00	0,00	1,00	2,00	3,00	1,94
Mécanismes de dialogue et de participation communautaire	5,00	1,00	3,00	3,00	1,00	0,00	2,00	2,00	2,00	4,00	3,00	2,00	3,00	1,00	0,00	2,00	3,00	2,18
Diffusion d'informations claires et adaptées	0,00	1,00	4,00	2,00	1,00	0,00	3,00	2,00	4,00	0,00	3,00	4,00	5,00	0,00	2,00	2,00	3,00	2,12
Score moyen	1,75	0,50	3,50	2,50	0,75	1,00	2,75	2,25	3,00	1,00	2,75	2,25	3,50	0,25	0,75	2,00	2,50	1,94

**Mesures de prévention et de contrôle:** seulement un district (5%) avait un niveau de préparation optimal (Mbanga). Trois districts (17%) avaient un bon niveau de préparation (Logbaba, Yabassi, Newbell). Tous les autres districts avaient de faibles niveaux de préparation allant du niveau insuffisant (41%) au niveau limité (35%). Le respect des mesures d’hygiène et de biosécurité était l´élément le plus significatif (préparation limitée) dans la région du Littoral, conduisant la région à un niveau de préparation limité pour cette dimension (Tableau 4).

**Tableau 4:** mesures de prévention et de contrôle

	Bangue	Boko	Edea	Logbaba	Manoka	Ndom	Ngambe	Pouma	Yabassi	Manjo	Newbell	Njombe penja	Mbanga	Nkondjock	Loum	Dibombari	Nkongsamba	Littoral
**Mesures de prévention et de contrôle**																		
Mise en place de mesures de contrôle et de prévention	0,00	1,00	2,00	4,00	1,00	0,00	1,00	0,00	4,00	0,00	3,00	3,00	5,00	0,00	0,00	2,00	3,00	1,71
Suivi des contacts et gestion des cas	3,00	0,00	1,00	3,00	0,00	0,00	2,00	0,00	4,00	0,00	5,00	1,00	5,00	0,00	0,00	2,00	0,00	1,53
Respect des mesures d'hygiène et de biosécurité	3,00	2,00	1,00	4,00	1,00	0,00	2,00	0,00	3,00	3,00	4,00	2,00	5,00	0,00	1,00	1,00	3,00	2,06
Score moyen	2,00	1,00	1,33	3,67	0,67	0,00	1,67	0,00	3,67	1,00	4,00	2,00	5,00	0,00	0,33	1,67	2,00	1,76
Score Global Moyen	2,26	1,21	2,11	2,16	0,95	0,63	2,32	1,05	3,00	1,53	3,21	2,26	3,16	0,16	0,74	1,79	2,16	1,80

**Coordination et gestion de la réponse:** les résultats montraient que 47% (8/17) des districts de santé avaient un niveau de préparation insuffisant, 29% (5/17) avaient un niveau de préparation limité, et un seul (5%) avait un bon niveau de préparation (Mbanga). Seul l´élément désignation d’un responsable/équipe de gestion de la riposte était moyennement préparé, tandis que l´allocation de ressources financières et matérielles suffisantes avait un niveau de préparation insuffisant dans tous les districts. Conduisant la région du Littoral à un niveau de préparation limité pour cette composante (Tableau 5).

**Tableau 5:** coordination et gestion de la réponse

	Bangue	Boko	Edea	Logbaba	Manoka	Ndom	Ngambe	Pouma	Yabassi	Manjo	Newbell	Njombe penja	Mbanga	Nkondjock	Loum	Dibombari	Nkongsamba	Littoral
**Coordination et gestion de la réponse**																		
Existence d'un plan de préparation et de riposte au Mpox	0,00	0,00	0,00	0,00	0,00	0,00	3,00	0,00	2,00	0,00	3,00	2,00	5,00	0,00	0,00	2,00	0,00	1,00
Mise en place d'un mécanisme de coordination multisectorielle	3,00	0,00	0,00	1,00	1,00	0,00	0,00	3,00	3,00	0,00	3,00	1,00	5,00	0,00	0,00	3,00	2,00	1,47
Désignation d'un responsable/équipe de gestion de la riposte	3,00	1,00	2,00	3,00	0,00	0,00	5,00	2,00	3,00	5,00	5,00	2,00	5,00	0,00	0,00	3,00	0,00	2,29
Allocation de ressources financières et matérielles suffisantes	0,00	0,00	0,00	0,00	0,00	0,00	1,00	0,00	1,00	0,00	0,00	0,00	0,00	0,00	0,00	0,00	0,00	0,12
Score moyen	1,50	0,25	0,50	1,00	0,25	0,00	2,25	1,25	2,25	1,25	2,75	1,25	3,75	0,00	0,00	2,00	0,50	1,22

**Prise en charge des patients:** la prise en charge des cas était la moins notée (1,07) dans la région avec un très faible niveau de préparation (préparation insuffisante). Seulement 11% (2/17) avaient un niveau de préparation moyen (Yabassi, Newbell) ; tous les autres districts avaient de faibles niveaux de préparation allant du niveau insuffisant au niveau limité. Les éléments « Disponibilité de protocoles de prise en charge clinique » et « Formation du personnel de santé sur la prise en charge » faisaient partis des éléments les moins préparés (préparation insuffisante) dans la région du Littoral toutes composantes confondues. Aucun district n´avait un niveau de préparation optimal (Tableau 6).

**Tableau 6:** prise en charge des cas

	Bangue	Boko	Edea	Logbaba	Manoka	Ndom	Ngambe	Pouma	Yabassi	Manjo	Newbell	Njombe penja	Mbanga	Nkondjock	Loum	Dibombari	Nkongsamba	Littoral
**Prise en charge des cas**																		
Disponibilité de protocoles de prise en charge clinique	0,00	0,00	1,00	0,00	0,00	0,00	1,00	0,00	4,00	0,00	3,00	4,00	0,00	0,00	0,00	2,00	0,00	0,88
Formation du personnel de santé sur la prise en charge	0,00	0,00	0,00	0,00	0,00	0,00	1,00	0,00	2,00	0,00	1,00	1,00	0,00	0,00	0,00	0,00	0,00	0,29
Disponibilité d'équipements de protection individuelle	3,00	5,00	2,00	3,00	5,00	0,00	0,00	2,00	3,00	0,00	3,00	2,00	0,00	0,00	4,00	0,00	3,00	2,06
Accès aux traitements et aux soins de santé adéquats	3,00	0,00	2,00	2,00	0,00	0,00	1,00	0,00	3,00	0,00	3,00	1,00	0,00	0,00	0,00	0,00	3,00	1,06
Score moyen	1,50	1,25	1,25	1,25	1,25	0,00	0,75	0,50	3,00	0,00	2,50	2,00	0,00	0,00	1,00	0,50	1,50	1,07

**Niveau de préparation globale et la résilience:** la région du Littoral avait un niveau de préparation limité considérant que 5 districts (29%) avaient une préparation insuffisante (Nkondjock, Loum, Manoka, Ndom, Pouma) ; 17% (3/17) des districts avaient une préparation limitée (Boko, Manjo, Dibombari); 41% (7/17) avaient une préparation moyenne (Bangue, Edea, Logbaba, Ngambe, Yabassi, Njombe Penja, Nkongsamba) et seulement 11% (2/17) avaient un bon niveau de préparation (Newbell, Mbanga). Aucun district de la région du Littoral n´avait un niveau de préparation optimal. En qualité de résilience communautaire les mécanismes de dialogue et de participation communautaire avaient un niveau de préparation moyen (Tableau 7).

**Tableau 7:** niveau de préparation globale et la résilience

	Bangue	Boko		Edea	Logbaba	Manoka	Ndom	Ngambe	Pouma	Yabassi	Manjo	Newbell	Njombe penja	Mbanga	Nkondjock	Loum	Dibombari	Nkongsamba	Littoral
**Coordination et gestion de la réponse**																			
Score moyen	1,50	0,25		0,50	1,00	0,25	0,00	2,25	1,25	2,25	1,25	2,75	1,25	3,75	0,00	0,00	2,00	0,50	1,22
**Surveillance épidémiologique et laboratoire**																			
Score moyen	4,50	3,00		3,75	2,75	1,75	2,00	4,00	1,00	3,25	4,25	4,25	3,75	4,00	0,50	1,50	2,75	4,25	3,01
**Prise en charge des cas**																			
Score moyen	1,50	1,25		1,25	1,25	1,25	0,00	0,75	0,50	3,00	0,00	2,50	2,00	0,00	0,00	1,00	0,50	1,50	1,07
**Communication des risques et engagement communautaire**																			
Score moyen	1,75	0,50		3,50	2,50	0,75	1,00	2,75	2,25	3,00	1,00	2,75	2,25	3,50	0,25	0,75	2,00	2,50	1,94
**Mesures de prévention et de contrôle**																			
Score moyen	2,00	1,00	1,33		3,67	0,67	0,00	1,67	0,00	3,67	1,00	4,00	2,00	5,00	0,00	0,33	1,67	2,00	1,76
Score Global Moyen	2,26	1,21	2,11		2,16	0,95	0,63	2,32	1,05	3,00	1,53	3,21	2,26	3,16	0,16	0,74	1,79	2,16	1,80

## Discussion

L’évaluation de la préparation des districts de santé de la région du Littoral face à une éventuelle épidémie de mpox a mis en évidence des vulnérabilités significatives qui pourraient compromettre la capacité du système de santé à détecter rapidement les cas, à limiter la transmission et à fournir des soins de qualité aux personnes infectées. Cette étude va dans le sens de celle menée au Nigéria (2023) qui visait à présenter plusieurs préoccupations urgentes concernant l´état de préparation du secteur de la santé Nigérian face à la réémergence du virus de la variole du singe et la réponse à cette épidémie [3].

Une étude menée à l’échelle continentale en 2022 a révélé des lacunes significatives dans la préparation des pays africains face à une épidémie de mpox. Les systèmes de surveillance étaient souvent défaillants, avec des données incomplètes, des analyses tardives et un accès limité aux outils de diagnostic et de traitement [14]. En parallèle, notre étude a mis en évidence un niveau de préparation moyen, avec des forces dans certains domaines comme l’analyse de données et la définition de cas. Ceci pourrait s´expliquer par la mise en place de systèmes de surveillance épidémiologique actifs qui permettent une détection précoce des cas. Cela inclut la collecte de données et le suivi des maladies dans les districts. De plus, la tenue hebdomadaire des réunions de surveillance de routine avec le Centre Régional de Prévention et de Lutte contre les Epidémies (CERPLE) où sont formés de nombreux professionnels de la santé des districts de santé de la région sur les définitions des cas des maladies sous surveillance épidémiologique, améliorent la capacité de réponse face aux épidémies et facilitent la notification et la remontée des cas. Aussi, la région bénéficie souvent de l´appui d´organisations internationales et de partenaires de développement qui fournissent des ressources pour la notification des cas et des formations. C´est ainsi qu´au Nigéria, la mise en place d´une réponse efficace à l´épidémie de variole du singe dépendait en grande partie de la formation adéquate du personnel de santé, des épidémiologistes, des biostatisticiens [15].

Les maladies infectieuses comme la variole du singe nécessitent des capacités de laboratoire en matière de réaction en chaîne par polymérase [16]. Cependant, les résultats de cette étude montrent qu´il existe des faiblesses persistantes, notamment en matière de diagnostic ; car aucun district de la région ne disposait ni de tests de diagnostic rapide (TDR), ni de la détection de l´ADN viral par amplification en des chaînes par polymérase (PCR) qui est le test de laboratoire à privilégier pour la mpox [17]; ceci pourrait favoriser la propagation de la maladie, la difficulté à identifier les cas suspects, les limitations dans la surveillance épidémiologique, la surcharge des systèmes de santé, la perte de confiance du public et les difficultés dans la planification sanitaire. Ces deux études s’accordent sur l’importance cruciale d’une surveillance solide pour une réponse efficace aux épidémies. Les disparités observées mettent en évidence la nécessité d’adapter les stratégies de renforcement des systèmes de santé à chaque contexte tout en tirant parti des bonnes pratiques existantes. Dans le même ordre d´idée, les résultats d´une étude ont montré que le manque d’installations diagnostiques adéquates avait grandement entravé la réussite à la réponse de l’épidémie de mpox. Il est donc plausible et logique que l´amélioration des capacités de diagnostic soit une priorité [16].

Une étude menée au Royaume-Uni (2023) a révélé que l’implication précoce de la communauté, facilitée par la direction de l’essai et les bailleurs de fonds, avait joué un rôle clé dans la réponse au mpox, grâce à un comité consultatif communautaire qui avait aidé à l’élaboration du protocole et au recrutement [18]. La présente étude quant à elle a démontré des niveaux de préparation variés, avec seulement 11% des districts montrant une bonne préparation. Bien que les mécanismes de dialogue communautaire et la diffusion d’informations claires avaient le meilleur niveau de préparation, le niveau de préparation concernant l’élaboration d’un plan de communication des risques était insuffisant. Les travaux d´une étude menée aux Etats-Unis (2022) ont démontré que la stigmatisation, en influençant les politiques de santé et l’accès aux soins, peut entraver la lutte contre les maladies. Cependant, des campagnes d’éducation ciblées peuvent inverser cette tendance en favorisant la recherche de soins et en améliorant la perception de la maladie [19]. Au Nigéria (2019), dès le début de l´épidémie de variole du singe, la faible sensibilisation à l´épidémie aurait eu un impact négatif sur la réponse [20].

En République du Congo, des recherches ont montré que les gens croyaient beaucoup à l´introduction artificielle du virus dans la communauté. En même temps, certains ne croyaient pas à l´existence du virus [21]. Toujours au Nigéria, une étude a révélé la prévalence des croyances conspirationnistes au sujet de la variole du singe parmi les habitants du Sud-Sud et du Sud-Est [22]. Une diffusion accrue de connaissances exactes provenant de sources crédibles par le biais de médias tels que les journaux, la radio et les panneaux d´affichage contribuera à accroître les niveaux de connaissance de la maladie [23,24]. En investissant dans la communication des risques, l’engagement communautaire et la coordination intersectorielle, il est possible de développer des réponses plus adaptées et plus efficaces, tout en favorisant l’apprentissage continu et l’amélioration des pratiques.

L’hygiène joue un rôle central dans la prévention de la propagation du virus. Des mesures rigoureuses, telles que le nettoyage et la désinfection réguliers des environnements contaminés, associées à un dépistage systématique et à l’isolement des animaux infectés, permettraient de limiter considérablement la transmission du virus et d’éviter l’apparition de nouveaux foyers [25]. Certains travaux ont ressorti l’indisponibilité d’infrastructures d’isolement dans les hôpitaux [15]. Notre évaluation des mesures de prévention et de contrôle de la variole du singe dans la région du Littoral a révélé des disparités significatives entre les districts, avec un niveau global de préparation jugé insuffisant. Bien que les pratiques d’hygiène et de biosécurité aient été relativement bien mises en œuvre, d’autres composantes du dispositif de préparation, telles que la surveillance épidémiologique et la capacité de réponse, présentaient des lacunes. Ces résultats sont en partie concordants avec ceux de plusieurs études menées en Chine (2022) et au Nigéria (2020) qui faisaient ressortir l’importance de stratégies de contrôle ciblées, notamment la recherche des contacts dans les populations à risque [26-27]. Il est nécessaire de mettre l’accent sur le strict respect des mesures de sécurité sanitaire afin de limiter la propagation de la variole du singe à l’échelle nationale [3]. Pour renforcer la résilience des districts de santé de la région face aux menaces sanitaires émergentes, il est impératif d’élargir la portée des mesures de prévention et de contrôle, en mettant l’accent sur le développement de plans d’urgence adaptés aux spécificités locales et en investissant dans la formation du personnel de santé.

Nos résultats, tout comme ceux de l´étude menée par le bureau de la responsabilité gouvernementale aux États-Unis (2024), mettent en évidence un déficit significatif en matière de préparation aux épidémies en ce qui concerne le volet coordination et gestion de la réponse [28]. Seul le district de santé de Mbanga avait un bon niveau de préparation. Ce district est limitrophe des districts de santé de la région anglophone. En effet, la région anglophone demeure le foyer principal des cas au total de la maladie depuis janvier 2023, où des cas de mpox avaient été détectés dans les districts de santé de Mbonge et de Kumba [29]. Tout ceci a certainement servi d´expérience au district de santé de Mbanga afin d´améliorer sa préparation face à une éventuelle épidémie de mpox.

Si notre étude révèle des lacunes au niveau local des districts de santé de la région du Littoral, l’étude américaine souligne, quant à elle, un manque de coordination au niveau fédéral. Bien que les contextes diffèrent, ces deux études convergent vers un constat alarmant: la nécessité d’améliorer considérablement les systèmes de préparation aux urgences sanitaires, tant au niveau national qu’au niveau local. Elles soulignent toutes deux l’importance d’une coordination renforcée entre les différents acteurs et de l’établissement de plans de préparation clairs et régulièrement actualisés. Elles vont dans le même sens que l´étude menée au Nigéria (2023) où le système de surveillance, de réponse, de gestion et d’analyse des épidémies avait été créé par le Centre Nigérian de Contrôle des Maladies et le Centre national d´opérations d´urgence multisectorielles pour la variole du singe avait été activé afin d’améliorer encore la rapidité des rapports et des réponses [3]. Ces initiatives avaient renforcé le contrôle de la propagation de la variole du singe [16,28-29]. Aussi, pour renforcer encore davantage ses capacités, le « Centre national multisectoriel d’opérations d’urgence pour la variole du singe » avait été activé le 26 mai 2022 afin de coordonner et de renforcer les réponses et les activités dans le pays et, en même temps, de contribuer à la lutte mondiale [30].

Les résultats d´une étude menée en Malaisie (2022) ont montré que la prise en charge des cas de variole du singe était principalement centrée sur les soins locaux des lésions cutanées, tout en s’appuyant sur un système de surveillance épidémiologique relativement développé, notamment grâce à l’utilisation de l’application MySejahtera [13]. Au Nigéria, le Centre nigérian de contrôle des maladies avait élaboré un protocole de surveillance active de l’orthopoxvirose simienne chez l’animal ciblant les zones à haut risque à l’interface homme-animal [28,29].

En revanche, notre étude a mis en évidence des carences significatives dans la préparation des districts de santé du Littoral à faire face à une telle épidémie. Les protocoles clairs et standardisés pour la gestion des cas de mpox étaient peu nombreux et le personnel de santé manquait de formation sur la prise en charge de cette maladie. Ces disparités illustrent les défis inégaux auxquels sont confrontés différents contextes géographiques en matière de préparation aux maladies émergentes.

Il convient de noter que cette étude présente certaines limitations liées à sa nature transversale, qui ne permet pas d’étudier l’évolution des pratiques dans le temps. De plus, l’évaluation a été réalisée auprès des responsables des équipes cadres, ce qui pourrait introduire un biais de perception. Des études complémentaires, telles que des enquêtes auprès de différents acteurs et une surveillance continue, seraient nécessaires pour affiner ces résultats et mieux comprendre la dynamique de la préparation dans la région.

## Conclusion

La région du Littoral a un niveau de préparation limité, avec des lacunes importantes dans certains domaines clés. Afin de prévenir une éventuelle épidémie de mpox dans la région du Littoral, il est essentiel de renforcer la préparation des districts sur la coordination et gestion de la réponse, la prise en charge des cas, la communication des risques et engagement communautaire, les mesures de prévention et de contrôle en intégrant une approche différenciée tenant compte des disparités entre districts.

## Remerciements

Les auteurs remercient les responsables des équipes cadres des districts de santé de la région du Littoral Cameroun pour leur disponibilité lors de cette évaluation.
